# Nrf2/ARE Signaling Directly Regulates SOX9 to Potentially Alter Age-Dependent Cartilage Degeneration

**DOI:** 10.3390/antiox11020263

**Published:** 2022-01-28

**Authors:** Yusuke Kubo, Rainer Beckmann, Athanassios Fragoulis, Claudius Conrads, Prathyusha Pavanram, Sven Nebelung, Michael Wolf, Christoph Jan Wruck, Holger Jahr, Thomas Pufe

**Affiliations:** 1 Department of Anatomy and Cell Biology, Uniklinik RWTH Aachen, Wendlingweg 2, D-52074 Aachen, Germany; rainer.beckmann@wiwi.uni-kl.de (R.B.); afragoulis@ukaachen.de (A.F.); claudius.conrads@rwth-aachen.de (C.C.); ppavanram@ukaachen.de (P.P.); cwruck@ukaachen.de (C.J.W.); hjahr@ukaachen.de (H.J.); tpufe@ukaachen.de (T.P.); 2 Department of Diagnostic and Interventional Radiology, Uniklinik RWTH Aachen, Pauwelsstraße 30, D-52074 Aachen, Germany; snebelung@ukaachen.de; 3 Department of Orthodontics, Uniklinik RWTH Aachen, Pauwelsstraße 30, D-52074 Aachen, Germany; michwolf@ukaachen.de; 4 Department of Orthopaedic Surgery, Maastricht University Medical Center+, 6229 HX Maastricht, The Netherlands

**Keywords:** SOX9, Nrf2, antioxidant response element, Keap1, osteoarthritis

## Abstract

Oxidative stress is implicated in osteoarthritis, and nuclear factor erythroid 2–related factor 2 (Nrf2)/antioxidant response element (ARE) pathway maintains redox homeostasis. We investigated whether Nrf2/ARE signaling controls SOX9. SOX9 expression in human C-28/I2 chondrocytes was measured by RT–qPCR after shRNA-mediated knockdown of Nrf2 or its antagonist the Kelch-like erythroid cell-derived protein with cap ‘‘n’’ collar homology-associated protein 1 (Keap1). To verify whether Nrf2 transcriptionally regulates SOX9, putative ARE-binding sites in the proximal *SOX9* promoter region were inactivated, cloned into pGL3, and co-transfected with phRL–TK for dual-luciferase assays. *SOX9* promoter activities without and with Nrf2-inducer methysticin were compared. Sox9 expression in articular chondrocytes was correlated to cartilage thickness and degeneration in wild-type (WT) and Nrf2-knockout mice. Nrf2-specific RNAi significantly decreased *SOX9* expression, whereas Keap1-specific RNAi increased it. Putative ARE sites (ARE_1_, ARE_2_) were identified in the *SOX9* promoter region. ARE_2_ mutagenesis significantly reduced *SOX9* promoter activity, but ARE_1_ excision did not. Functional ARE_2_ site was essential for methysticin-mediated induction of *SOX9* promoter activity. Young Nrf2-knockout mice revealed significantly lower Sox9-positive chondrocytes, and old Nrf2-knockout animals showed thinner cartilage and more cartilage degeneration. Our results suggest Nrf2 directly regulates SOX9 in articular cartilage, and Nrf2-loss can develop mild osteoarthritis at old age. Pharmacological Nrf2 induction may hold the potential to diminish age-dependent cartilage degeneration through improving SOX9 expression.

## 1. Introduction

Osteoarthritis (OA), the most common joint disease, is characterized by cartilage damage and joint dysfunction [1]. Altered chondrocyte behavior such as chondrocyte hypertrophy, matrix ossification, and osteophyte formation in the joint is closely connected to its pathogenesis [2]. SOX9 is an essential transcription factor controlling cartilage extracellular matrix (ECM) homeostasis and a key regulator of chondrocyte differentiation through promoting collagen type II (Col II) expression [3]. Consequently, SOX9 inactivation before mesenchymal condensation downregulated Col II expression, to affect cartilage formation in a mouse model [4]. Previously, a reduced SOX9 gene expression in articular chondrocytes from osteoarthritic patients was also reported [5]. Since SOX9 directly represses Runx2, a key regulator of OA development and progression through BapX1 [5,6], it is a dominant, negative regulator of chondrocyte hypertrophy [7]. The induction of SOX9 seems to have the potential to prevent ECM degradation by inhibiting RUNX2 and, therefore, constitutes a therapeutic target for slowing the progression of OA.

Oxidative stress has also been increasingly recognized as closely tied to OA pathology [8]. Oxidative stress is generally defined as an imbalance between reactive oxygen species (ROS) production and cell defense system by antioxidants. Overproduced ROS are involved in the regulation of various biochemical factors associated with joint inflammation or cartilage degradation. Furthermore, ROS reduce the synthesis of ECM components by inducing chondrocyte cell death and activating latent metalloproteinases [9]. Nuclear factor erythroid 2–related factor 2 (Nrf2) crucially controls the antioxidant response and is essential for maintaining cellular redox homeostasis, as it attaches to the antioxidant response element (ARE) to promote the expression of antioxidative stress enzymes [10]. In a physiological state, the Kelch-like erythroid cell-derived protein with cap ‘‘n’’ collar homology-associated protein 1 (Keap1) binds to Nrf2 and promotes its ubiquitination, subsequently leading to proteolysis of Nrf2 [11]. This sequestration and further degradation of Nrf2 in the cytoplasm is the inhibitory effect of Keap1 on Nrf2. Several Nrf2 inducers were previously established; Wruck et al. [10] demonstrated that kavalactones, such as methysticin, can attenuate amyloid beta-peptide toxicity through Nrf2-mediated induction of protective gene expression in vitro. This suggests that the use of purified kavalactones might be considered as an adjunct therapeutic strategy to combat oxidative stress-related diseases. Previous studies have further shown that Nrf2 deficiency can cause severe OA in a murine drug-induced arthritis model or joint instability model due to meniscus removal [12,13]. Seemingly, Nrf2 is also capable of affecting chondrocyte differentiation through Col II expression [6]; nevertheless, the nature of the relationship between Nrf2 and SOX9 in chondrocytes remains unclear.

Therefore, the purpose of this study was to investigate (i) whether Nrf2/ARE signaling can directly regulate SOX9 expression in hyaline cartilage and (ii) if chronic Nrf2 deficiency may contribute to age-dependent articular cartilage degeneration.

## 2. Materials and Methods

### 2.1. Cell Culture

We used human C-28/I2 chondrocytes immortalized with Simian virus 40 large T antigen (SV40-TAg), as a well-established model phenotypically resembling articular chondrocytes [14,15]. This cell line was a kind gift from Dr. Mary Goldring. Cells were seeded at a density of 100,000/cm^2^, in 75 cm^2^ culture flasks, and cultured in Dulbecco’s modified Eagle medium/nutrient mixture F-12 (DMEM/F12) medium containing 10% heat-inactivated fetal calf serum (FCS), 100 U/mL penicillin–streptomycin, and 2.5 μg/mL amphotericin B. The medium was changed three times per week. All media and culture additives were purchased from Merck (Darmstadt, Germany) or Thermo Fisher Scientific (Dreieich, Germany).

### 2.2. RNA Interference (RNAi)

All short hairpin RNAs (shRNA) were purchased from Merck (Darmstadt, Germany) and were part of their MISSION^®^ shRNA library. Lentiviral particles were generated to transduce target cells as described before [16]. The expression of Nrf2 and Keap1 in C28/I2 chondrocytes was inhibited by two different shRNA constructs each and was used with the commercial non-target control (NTC) shRNA construct, 5′-CCGGCGCGATAGCGCTAATAATTTCTCGAGAAATTATTAGCGCTATCGCGCTTTTT-3′ (Cat. #SHC016, Merck, Darmstadt, Germany). The shRNA sequences were as follows: Nrf2 shRNA-1 (TRC clone ID: TRCN0000007558), 5′-CCGGCCGGCATTTCACTAAACACAACTCGAGTTGTGTTTAGTGAAATGCCGGTTTTT-3′; Nrf2 shRNA-2 (TRC clone ID: TRCN0000007555), 5′-CCGGGCTCCTACTGTGATGTGAAATCTCGAGATTTCACATCACAGTAGGAGCTTTTT-3′; Keap1 shRNA-1 (TRC clone ID: TRCN0000154657), 5′-CCGGGCGAATGATCACAGCAATGAACTCGAGTTCATTGCTGTGATCATTCGCTTTTTTG-3′; Keap1 shRNA-2 (TRC clone ID: TRCN0000156676), 5′-CCGGGTGGCGAATGATCACAGCAATCTCGAGATTGCTGTGATCATTCGCCACTTTTTTG-3′.

### 2.3. Quantification of Gene Expression by RT-qPCR

RNA isolation for gene expression analyses was performed with PeqGold Trifast (Peqlab, Erlangen, Germany), based on the manufacturer’s protocols. Following isolation, the quantity and purity of the RNA were measured by spectrophotometry using a NanoDrop 1000 system (Peqlab, Erlangen, Germany) (calculation of A_260_/A_280_ & A_260_/A_230_ ratios). Furthermore, RNA integrity was determined by MOPS-buffered agarose gel electrophoresis (28S rRNA; 5 kb and 18S rRNA; 1.9 kb). Reverse transcription was performed with the Maxima First Strand cDNA synthesis kit according to the recommendations of the supplier (Thermo Fisher Scientific, Dreieich, Germany). qPCR was conducted on an ABI StepOne Plus system, using PowerSYBR^®^ Green PCR Master Mix (Thermo Fisher Scientific, Dreieich, Germany). Amplification efficiencies were calculated with the LinRegPCR program (Heart Failure Research Center, Amsterdam, The Netherlands) [17]. Gene expression levels of GOIs (gene of interests) were normalized to a reference gene index (RGI), which was evaluated prior to the qPCR study, using geNorm calculation. These analyses revealed that normalization using the geometric mean of gene expressions levels of glyceraldehyde dehydrogenase (GAPDH) and beta-actin (ACTB) would be optimal in this experimental setup. The relative gene expression levels of GOI were calculated with qbase+ 3.2 software (Biogazelle, Gent, Belgium). The primers human *SOX9* (Cat. #QT00001498, QIAGEN, Hilden, Germany), *Nrf2* (forward 5′-TCCAGTCAGAAACCAGTGGAT-3′ and reverse 5′-GAATGTCTGCGCCAAAAGCTG-3′) (Eurofins MWG Operon, Ebersberg, Germany), and *Keap1* (forward 5′-TATCCACCCCAAGGTCATGGA-3′ and reverse 5′-GACAGGTTGAAGAACTCCTCT-3′) were used. *GAPDH* (forward 5′-TGCACCACCAACTGCTTAGC-3′ and reverse 5′-GGCATGGACTGTGGTCATGAG-3′) (Eurofins MWG Operon, Ebersberg, Germany) and *ACTB* (forward 5′-CTGGAACGGTGAAGGTGACA-3′ and reverse 5′-AAGGGACTTCCTGTAACAACGCA-3′) (Eurofins MWG Operon, Ebersberg, Germany) were used.

### 2.4. Promoter–Reporter Constructs and Dual-Luciferase Reporter Assay

Within the human proximal *SOX9* promoter sequence (NM000346.3, NG_012490.1) ARE sequence was investigated in silico, and fragments were cloned into pGL3-basic luciferase reporter vector (Promega, Walldorf, Germany). We then constructed the *SOX9* promoter fragments for our functional analyses.

The obtained pGL3::*SOX9* promoter–reporter constructs were subsequently co-transfected with the constitutively active reporter *Renilla* luciferase vector phRL–TK (Promega, Walldorf, Germany) into C-28/I2 chondrocytes. In total, 10,000 cells per well were seeded into 96-well plates and gradually starved by first reducing the FCS concentration to 0.5% over 3 days. *SOX9* promoter activity was then evaluated in cells stimulated with 50 μM and 100 μM of methysticin for 6 and 12 h, to investigate *SOX9* promoter regulation by Nrf2 activation. The cells without methysticin treatment were used as control samples.

*SOX9* promoter activities were compared both between promoter fragments, as well as between scenarios with and without methysticin treatment (100 µM) for 12 h. Furthermore, the *SOX9* promoter activity in cells with Nrf2- and Keap1-KD was measured. The bioluminescence of both luciferases was measured with the GloMax^®^ 96 Microplate Luminometer (Promega, Walldorf, Germany). To standardize results, ratios between Firefly- and Renilla-derived luciferase signals were calculated.

### 2.5. Cell Proliferation and Viability

Cells transfected with non-target, Nrf2-, and Keap1 shRNAs were seeded into 96-well plates at a density of 6000 cells and with 100 μL cell culture medium per well. Cell proliferation was assessed at 0 h, 24 h, and 48 h of incubation by CyQUANT^®^ Cell Proliferation Assay (Thermo Fisher Scientific, Dreieich, Germany). In addition, cell metabolic activity and viability were evaluated at time points 0 h, 12 h, 24 h, and 48 h of incubation by using CellTiter 96^®^ AQueous Non-Radioactive Assay (MTS) (Promega, Walldorf, Germany). Both procedures were conducted in 96-well plates according to the manufacturer’s protocol, with seeding densities of 6000 cells/well. Colored formazan dye of MTS assay, indicating metabolically active cells, was quantified at 490 nm in a fluorescence microplate reader (Infinite M200, TECAN, Crailsheim, Germany). CyQUANT^®^ Cell Proliferation Assay (Thermo Fisher Scientific, Dreieich, Germany) was used for cells stimulated with 100 μM of methysticin for Nrf2 activation (Lkt Laboratories, St. Paul, MN, USA). Results are normalized to the value without methysticin treatment at each time point or to the NTC at 0 h, respectively.

### 2.6. Animal Studies

For the impact of chronic Nrf2 deficiency on articular cartilage, Nrf2-knockout (KO) and wild-type (WT) mice were evaluated by histology and immunohistochemistry. Nrf2-KO mice were kindly provided by Prof. Yuet Wai Kan, who generated these mice, as described before [18].

The WT control mice were littermates of the Nrf2-KO mice. All animals were centrally housed in the laboratory animal facility of the Uniklinik RWTH Aachen (Aachen, Germany) under specific pathogen-free conditions. Up to 5 animals were caged on a 12 h light–dark cycle, with free access to food and water. Once the animals reached the desired age (group 1: 11–12 weeks; group 2: 90 weeks), they were killed in accordance with the German Animal Protection Act (TierSchG) and in agreement with regulations of the European Animal Research Association (killing notification protocol No. 40108A4). Dissection of the hind limbs was performed post mortem.

### 2.7. Histology and Immunohistochemistry

After sacrifice, one of the femurs was fixed with 4% formalin for 24 h and subsequently decalcified with ethylenediaminetetraacetic acid (EDTA) solution for two weeks. The tissues were then embedded in paraffin and sectioned for immunohistochemical and histological analysis.

Sox9-positive chondrocytes in articular cartilage were evaluated in young mature adult mice. Paraffin-embedded tissue sections were deparaffinized with xylene and alcohol and subsequently treated with 3% hydrogen peroxide in 100% methanol, to remove cross-reaction. The sections were heated in a microwave oven for antigen retrieval and blocked with bovine serum albumin and distilled water, respectively. Immunohistochemistry was performed using rabbit primary antibody against Sox9 (1:1000 in Tris-buffered saline; tebu-bio: BS1597), and the tissue sections were incubated at 4 °C overnight. This procedure was followed by swine anti-rabbit biotin (1:1000 in Tris-buffered saline; DAKO: E0353) for Sox9 and then by detection through peroxidase-labeled streptavidin–biotin (DAKO: P0397) staining for 10 min. As a color reagent, 3-Amino-9-ethylcarbazol (AEC)-kit (Thermo Fisher Scientific, Dreieich, Germany) was used. Numbers of Sox9-positive chondrocytes and total cells were counted in murine articular knee cartilage. Subsequently, Sox9-positive chondrocyte counts per 1 mm^2^ and their ratios (out of total cell counts) were compared between the WT and Nrf2-KO mice.

Additionally, cartilage degeneration was evaluated using Safranin-O staining in young mature adult and old mice. Paraffin-embedded tissue sections were deparaffinized with xylene and alcohol. The tissues were stained with Weigert’s iron hematoxylin working solution for 10 min. After the wash in running tap water, they were stained with a fast green solution and rinsed quickly with 1% acetic acid solution. Finally, the tissues were stained with 0.1% Safranin-O solution. The severity of OA in articular cartilage of the distal femur and proximal tibia was evaluated using the Osteoarthritis Research Society International (OARSI) scoring system, described by Glasson et al. [19]. The scale consists of 0 to 6 points (0, normal cartilage; 0.5, loss of Safranin-O stain without structural changes; 1, small fibrillations without cartilage loss; 2, vertical clefts down to the layer just below the surface and some loss of surface lamina; 3−6, the extension of vertical tears/erosions to the calcified cartilage). Cartilage thickness and the mean OA score of femur and tibia were compared between WT and Nrf2-KO mice.

### 2.8. Statistical Analysis

Bartlett and Shapiro–Wilk tests were used to test for homoscedasticity and normal distribution, respectively. Parametric data were analyzed by one-way ANOVA or a Student’s *t*-test (two-group comparisons). For histological analysis, the two groups (WT vs. KO) were compared using the non-parametric Wilcoxon rank-sum test, due to the lack of normal distribution. The histopathological evaluation was independently and blindly performed by two observers. Values are expressed as means ± standard error of the mean (SEM). Statistical significance was set to *p* < 0.05. All statistical analyses were performed using GraphPad Prism (version 8.0.0, GraphPad Software, San Diego, CA, USA) or the JMP Pro 13 software package (SAS Institute, Cary, NC, USA).

## 3. Results

### 3.1. SOX9 Expression in Response to Nrf2/Keap1 RNAi

**Figure 1:**
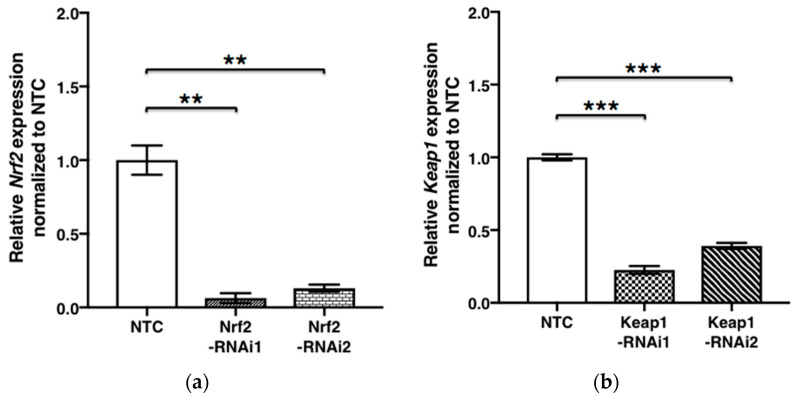

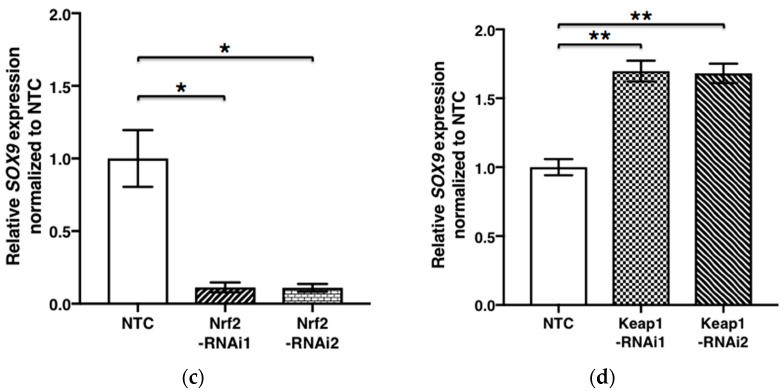
*SOX9* expression in chondrocytes is Nrf2/Keap1 dependent. *SOX9* expression in response to *Nrf2/Keap1* RNAi is shown. *Nrf2* (**a**), *Keap1* (**b**), and *SOX9* (**c**,**d**) expression upon RNA interference was quantified by RT–qPCR. Results were expressed relative to NTC (means ± SEM) (*n* = 3). ***, *p* < 0.0001; **, *p* < 0.001; *, *p* < 0.05 as indicated. NTC, non-target control; SEM, standard error of the mean.

Expression of human *Nrf2* (Gene ID: 4780) and *Keap1* (Gene ID: 9817) mRNA was effectively reduced by each knockdown with Nrf2- (*p* = 0.0005) and Keap1 shRNA (*p* < 0.0001). *SOX9* mRNA expression in chondrocytes transfected by Nrf2 shRNA was significantly lower than in NTC (*p* = 0.0060 and *p* = 0.0042). Conversely, *SOX9* (Gene ID: 6662) mRNA expression in cells transfected by Keap1 shRNA was significantly higher, compared with NTC (*p* = 0.0007) (Figure 1).

### 3.2. SOX9 Promoter–Reporter Constructs

**Figure 2:**
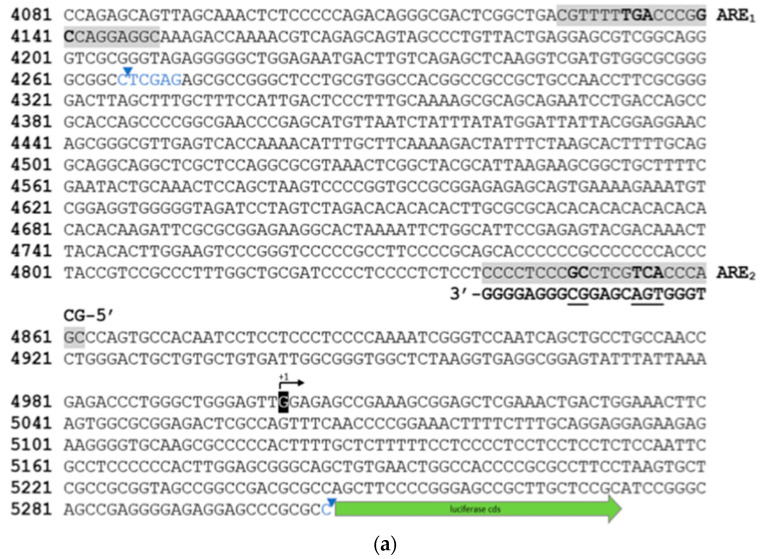

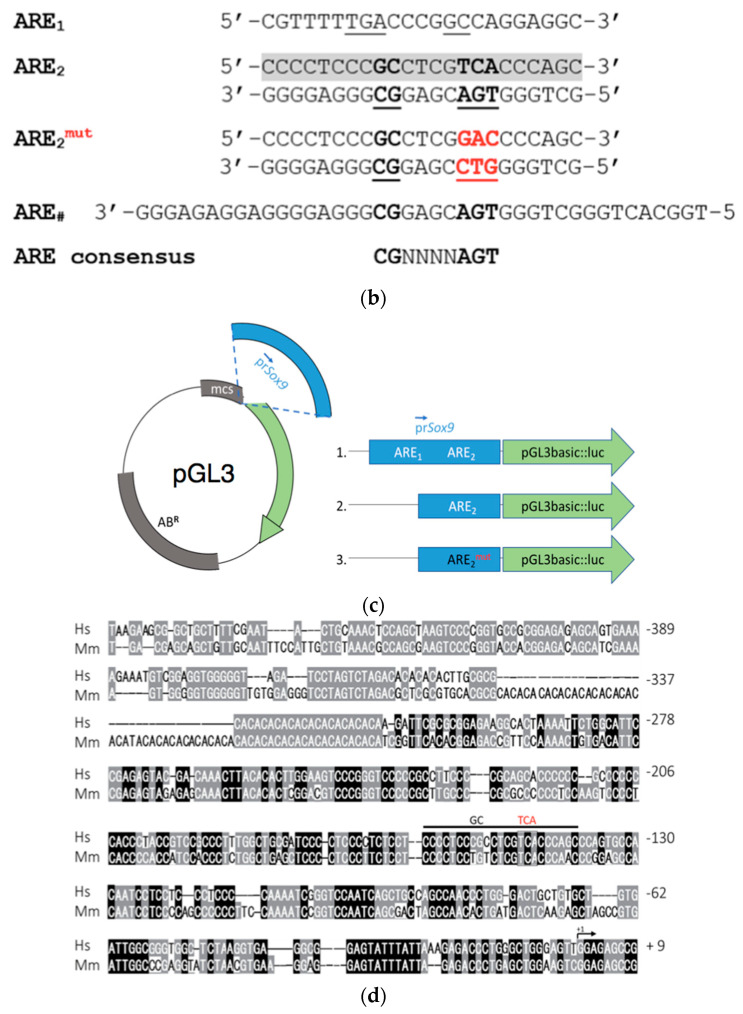
*SOX9* promoter–reporter constructs. Nucleotide sequence (5′-3′) fragment of the human *SOX9* promoter (NG_012490.1 nucleotide numbering indicated on left), indicating the relative position of two putative ARE sites (ARE_1_ and ARE_2_, respectively; color-marked in gray) and *Xho*I restriction sites indicated in blue (**a**). The transcription start site (TSS, +1) is indicated as G [20], and the coding sequence of the pGL3 luciferase gene is symbolized by a green arrow. Alignment of ARE_1_ and ARE_2_, mutated ARE_2_ (ARE_2_^mut^), and a recently published functional ARE site (ARE_#_ [21]) in the human *SOX9* promoter. ARE consensus sequence denotes the derived sequence [22], and letters in red indicate the site-directed mutation (**b**). Schematic of *SOX9* promoter (*prSOX9*, in blue) fragments, manipulation of ARE sites, and cloning strategy into pGL3-basic. Note that distal ARE_1_ sequence was truncated through *XhoI* restriction (*blue letters*; *SOX9*-ΔARE_1_), while proximal ARE_2_ sequence was mutated to result in *SOX9*-ΔARE_1_ARE_2_^mut^ (**c**). Human and mouse *SOX9*/*Sox9* nucleotide sequences (BLASTN search [23]) were aligned using Vector-NTI software (Thermo Fisher Scientific, Dreieich, Germany), to show conservation of ARE sites in the *Sox9* promoters between species. Location of functional ARE_2_ sites in promoter regions of both species is indicated with a horizontal black bar, between −113 and −115 from TSS (+1), with divergences from ARE consensus as introduced through mutation indicated by “GC” and “TCA” (in red) (**d**). ARE, antioxidant response element; +1, TSS; Hs, human; Mm, mouse; AB^R^, antibiotic resistance marker; mcs, multiple cloning site.

We identified two putative ARE sequences within the human proximal *SOX9* promoter sequence, ARE_1_ (5′-CGTTTT**TGA**CCCG**GC**CAGGAGG-3′) and ARE_2_ (5′-CCCTCCC**GC**CTCG**TCA**CCCAGC-3′) in silico and cloned fragments, −1034/+67 relative to its transcriptional start site (TSS), into pGL3-basic luciferase reporter vector. We then constructed three *SOX9* promoter fragments: (i) a full-length promoter fragment including both ARE_1_ and ARE_2,_ sites (referred to as *SOX9*); (ii) a fragment in which the distal ARE_1_ sequence was truncated using *Xho*I restriction (i.e., *SOX9*-ΔARE_1_); (iii) a fragment in which the proximal upstream ARE_2_ sequence was inactivated by point mutation (i.e., *SOX9*-ΔARE_1_ARE_2_^mut^) (Figure 2).

### 3.3. SOX9 Expression in Response to SOX9 Promoter Activity

**Figure 3:**
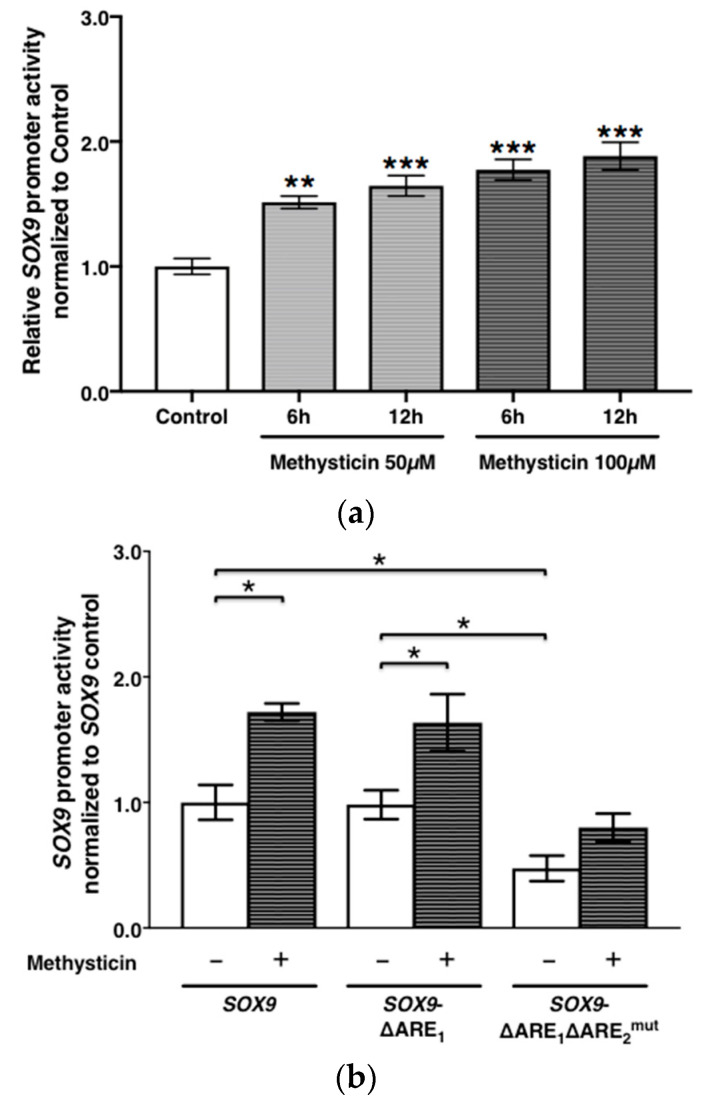

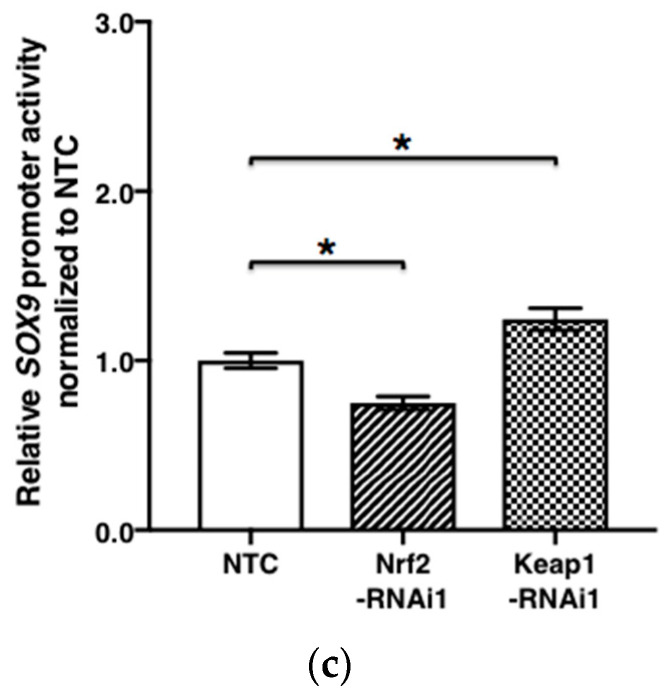
*SOX9* expression in response to *SOX9* promoter activity is shown. *SOX9* promoter activity 6 or 12 h after methysticin treatment (50 and 100 μM) was compared to without treatment (*n* = 8) (**a**). The promoter activities of *SOX9, SOX9*-ΔARE_1_, and *SOX9*-ΔARE_1_ARE_2_^mut^ were compared between treatment without and with 100 μM of methysticin (*n* = 4). Luciferase signals are normalized to reference signals (phRL–TK), and the activity of the *SOX9* fragment was set to 1.0 (means ± SEM) (**b**). *SOX9* promoter activity of transfected cells by Nrf2- and Keap1 shRNA was compared with the NTC (**c**). Results were expressed relative to NTC (means ± SEM) (*n* = 3). ***, *p* < 0.0001; **, *p* < 0.001; *, *p* < 0.05 as indicated.

The *SOX9* promoter activities 6 h and 12 h after 50 μM and 100 μM of methysticin were significantly higher than without treatment (50 μM-6 h, *p* = 0.0003; 50 μM-12 h and 100 μM-6 and −12 h, *p* < 0.0001, respectively). The *SOX9* promoter activity of the *SOX9*-ΔARE_1_ARE_2_^mut^ construct was significantly lower than that of either the full-length *SOX9* or the *SOX9*-ΔARE_1_ constructs, respectively (*p* = 0.0200). In contrast, there was no significant difference in *SOX9* promoter activities between the full-length *SOX9* fragment and *SOX9-*ΔARE_1_ constructs (*p* = 0.71). Methysticin treatment was associated with a significant increase in the *SOX9* promoter activities of the full-length *SOX9* (*p* = 0.0200), as well as the *SOX9*-ΔARE_1_ constructs (*p* = 0.0304), compared with non-treatment. However, *SOX9*-ΔARE_1_ARE_2_^mut^ promoter construct activity did not significantly respond to methysticin treatment (*p* = 0.0662). The *SOX9* promoter activity of cells transfected by Nrf2 shRNA was significantly lower than that in the NTC (*p* = 0.0042), while this activity of cells transfected by Keap1 shRNA was significantly higher than that in the NTC (*p* = 0.0051) (Figure 3).

### 3.4. Changes in Chondrocyte Proliferation and Metabolism upon Nrf2- and Keap1 RNAi

**Figure 4:**
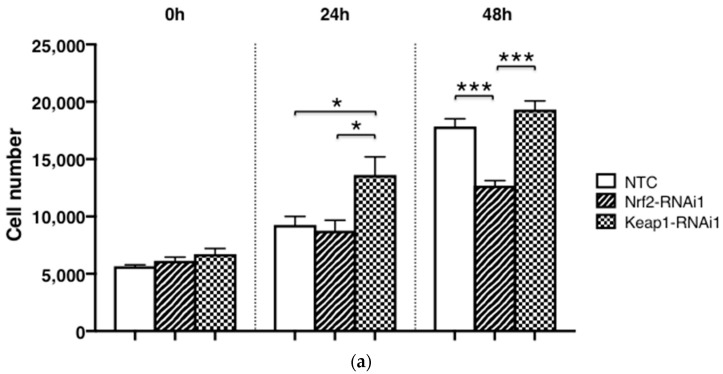

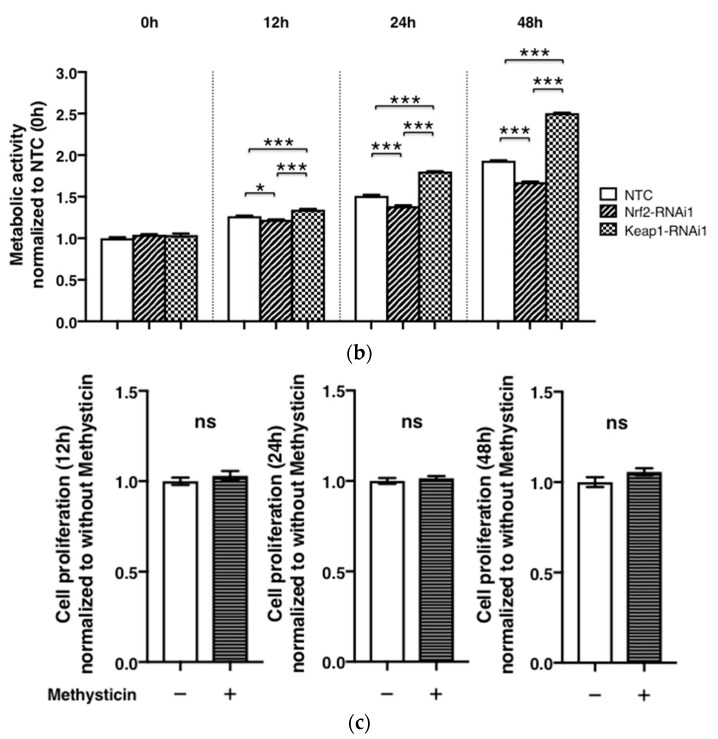
Chondrocyte proliferation is Nrf2- and Keap1-RNAi dependent but methysticin independent. Cell proliferation (**a**) and metabolic activity (**b**) of C28/I2 chondrocytes after RNA interference with Nrf2- and Keap1-specific shRNAs were compared with NTC at indicated time points post seeding (in hours). Results are expressed relative to NTC at 0 h (means ± SEM) (*n* = 4). Methysticin (100 μM) does not significantly affect cell proliferation (**c**). Results were normalized to the condition without methysticin treatment (*n* = 9). ***, *p* < 0.0001; *, *p* < 0.05; ns, not significant.

Already after 24 h, Keap1 knockdown significantly stimulated cell proliferation, compared with NTC and Nrf2 knockdown, respectively (Figure 4a, *p* < 0.05). In contrast, Nrf2 knockdown revealed a suppressive effect on cell proliferation, compared with NTC and Keap1 RNAi, respectively, which became highly significant at 48 h (*p* < 0.0001, Figure 4b). Transfecting chondrocytes with Keap1-specific shRNA, on the other hand, significantly increased their metabolic activity and viability, compared with the NTC (*p* < 0.0001). However, methysticin treatment did not significantly influence cell proliferation at any tested time point (12 h, *p* = 0.36; 24 h, *p* = 0.44; 48 h, *p* = 0.11, respectively) (Figure 4c).

### 3.5. Sox9 Staining in Hyaline Articular Cartilage of WT and Nrf2-KO Mice

**Figure 5:**
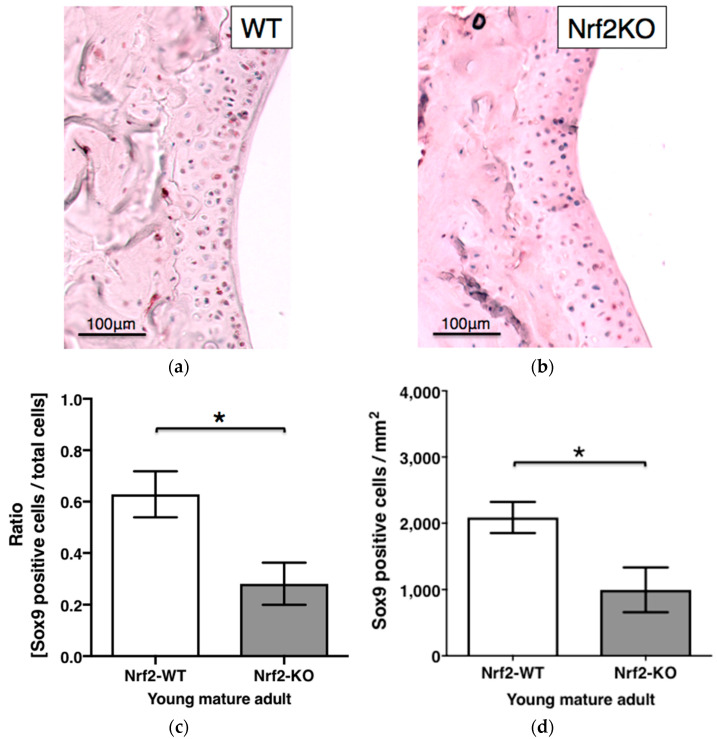
Sox9 staining in hyaline articular cartilage of WT and Nrf2-KO mice. Representative immunohistochemistry of articular cartilage of a murine knee showing Sox9 staining in young mature adult WT (*n* = 5) (**a**) and Nrf2-KO (**b**) mice (*n* = 6). Sox9-positive articular chondrocytes were counted and expressed as ratios (**c**) and cell number per 1 mm^2^ area (**d**) in WT and Nrf2-KO mice, respectively. Plotted are means ± SEM. *, *p* < 0.05; WT, wild type; KO, knockout.

The ratio of Sox9-positive chondrocytes in young mature adult Nrf2-KO mice (0.281 ± 0.082) was significantly lower than that in WT mice (0.629 ± 0.089) (*p* = 0.0446). Notably, Nrf2-KO mice expressed significantly less Sox9-positive chondrocytes in their articular cartilage than WT mice (*p* = 0.0446) (Figure 5).

### 3.6. Nrf2 Deficiency Causes Age-Dependent Cartilage Deterioration

**Figure 6:**
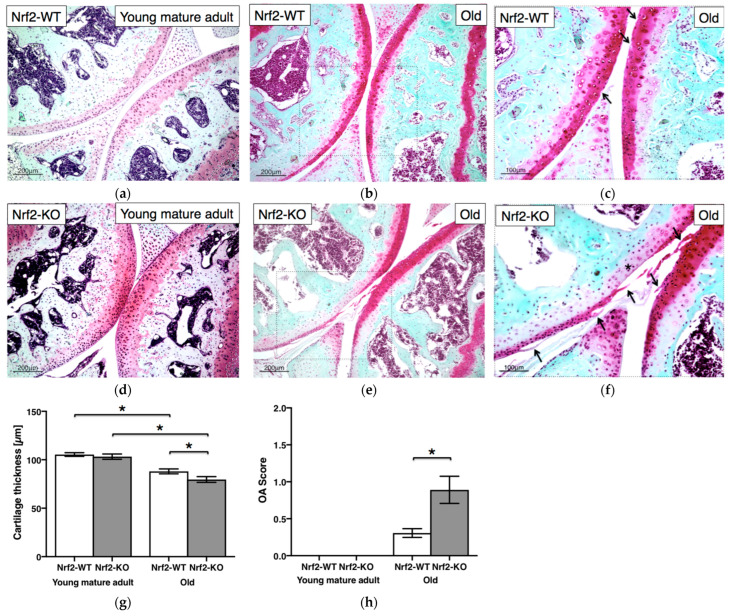
Nrf2 deficiency causes age-dependent cartilage deterioration. Representative histology of Safranin-O stained sections of articular knee cartilage from young mature adult (*n* = 6) (**a**) and old WT (*n* = 11) (**b**,**c**) and young mature adult (*n* = 5) (**d**) and old Nrf2-KO (*n* = 8) (**e**,**f**) mice. Cartilage thickness (μm) (**g**) and OA scores (**h**) were measured. Interestingly, cartilage erosion (asterisk) and surface fibrillation (i.e., fissures, arrows) were specifically observed only in old Nrf2-KO mice (OARSI grade: Femur 2 and Tibia 1) (**f**), while only loss of staining (i.e., arrows) was observed in old WT mice (OARSI grade: Femur 0.5 and Tibia 0.5) (**c**). Values are means ± SEM. *, *p* < 0.05. OA, osteoarthritis.

There was no significant difference in cartilage thickness between young mature adult WT mice (105.4 ± 1.9 μm) and Nrf2-KO (103.2 ± 2.8 μm; *p* = 0.58) mice, respectively (Figure 6a,d). In contrast, cartilage thickness in old Nrf2-KO mice (79.6 ± 3.0 μm) was significantly reduced, compared with that of WT littermates (88.0 ± 2.5 μm; *p* = 0.0454) (Figure 6b,e). Not surprisingly, overall cartilage thickness also decreased significantly with age, in both WT and Nrf2-KO mice (*p* = 0.0020 and *p* = 0.0043, respectively) (Figure 6g). Neither WT nor Nrf2-KO mice revealed osteoarthritic changes of their knee cartilage at young mature adult age, but rather at old age (magnified in Figure 6c,f). It is worth noting the superficial layer fissuring (arrows) and cartilage surface erosions (*) as osteoarthritic features. OA score of old Nrf2-KO mice (0.89 ± 0.18) was significantly higher than that of WT animals (0.31 ± 0.06; *p* = 0.0123), respectively (Figure 6h). The respective OA score for aged WT and Nrf2-KO mice is also summarized in Table 1.

## 4. Discussion

Due to the increasing awareness of the importance of oxidative stress to skeletal tissue integrity, and the importance of Sox9 to cartilage homeostasis, we investigated whether Nrf2/ARE signaling regulates SOX9 expression in hyaline articular cartilage.

First, we showed that methysticin, a well-established Nrf2 inducer, upregulated *SOX9* expression in chondrocytes. In silico, we then identified two putative Nrf2-binding sites (ARE_1/2_) in the *SOX9* proximal promoter region and, for the first time, demonstrated that ARE_2_ is crucial for the Nrf2-mediated *SOX9* induction in chondrocytes, through studying its mRNA expression and using dual-luciferase reporter gene assays. Successive inactivation of both putative ARE sites in the proximal human *SOX9* promoter region revealed that only mutagenesis of ARE_2_ reduced its promoter activity by approximately 50%. Our findings thus strongly indicate that ARE_2_ is a positive direct regulator of *SOX9* promoter activity in human chondrocytes. Notably, our effective concentration of methysticin did not significantly affect chondrocyte proliferation in vitro. Recently, Schmidlin et al. [21] reported a 41 bp functional ARE site in the *SOX9* promoter region of human non-small cell lung cancer cell line H1299. When comparing ARE_1_ and ARE_2_ sites from our study with those identified in H1299 cells, it appeared that our ARE_2_ on the Crick strand is identical with the functional ARE site in the *SOX9* promoter of H1299 cells. Furthermore, both core sequences match the ARE consensus site TGA NNN NGC [22]. As mutating ARE_2_ suppressed *SOX9* promoter activity in chondrocytes, this strongly suggests that this proximal ARE_2_ site is functional and crucial to the activation of the Nrf2 target gene in human chondrocytes.

Further, a successful, direct pharmacological activation of Nrf2 target genes by methysticin has already been shown in vitro and in vivo in another context [24,25]. Interestingly, Cao et al. [26] reported that cyanidin-induced Nrf2 overexpression reduced the relative mRNA expression of Sox9 and Col II in the murine embryonic mesenchymal progenitor cell line C3H10T1/2. At first glance, these data seemingly contradict our findings. However, the glycoside cyanidin also appears to be a potent sirtuin 6 activator, which is known to prevent, among others, chondrocyte senescence [27]. Cyanidin might have immediate but largely Nrf2-independent, and thus different, direct effects on C3H10T1/2 cells rather than on human coastal chondrocytes.

Notably, Sox9 is an essential transcription factor for the differentiation of the chondrocyte lineage during embryonic development, but also postnatally in the growth plate and articular chondrocytes alike [28]. Importantly, SOX9-mediated responses are well known to be largely context dependent [28]. Since Cao et al. used murine progenitor cells [26], while we used adult human chondrocytes, contrasting responses may result from differences in species and developmental stages as well.

In order to confirm our initial results using methysticin to pharmacologically stimulate Nrf2, we next used an Nrf2 knockdown-based approach in human chondrocytes. Nrf2-specific RNAi reduced *SOX9* promoter activity and mRNA expression in human chondrocytes in vitro. This is in line with results by Cheng et al. [29], reporting suppressed Sox9 expression upon siRNA-mediated downregulation of Nrf2 in the murine chondrogenic cell line ATDC5 [30]. Additionally, Nrf2 RNAi led to a significant reduction in cell proliferation and metabolic activity, which is in agreement with Sox9 being able to stimulate chondrocyte proliferation through, e.g., FGF-2 [31].

Not surprisingly, RNAi of Nrf2 antagonist Keap1, in contrast, resulted in higher promoter activity and mRNA expression of *SOX9* in chondrocytes in our hands. This is further circumstantial evidence that Nrf2 activation, through a loss of function of its antagonist Keap1, holds the potential to upregulate SOX9 expression in articular cartilage. Recent advances, furthermore, revealed that Keap1 contains multiple stress sensors, allowing it to respond to diverse cellular inputs, from oxidative stress through cellular metabolites to dysregulated autophagy, in order to regulate Nrf2 activity [32]. Considering that Nrf2 is a master regulator of ROS metabolism, as a future task, it is necessary to investigate whether possible alterations in redox balance could be involved in these effects in our study.

Another recent in vitro study also confirmed antagonistic actions and chondroprotective effects of Keap1 and the Nrf2 pathway in osteoarthritic chondrocytes [33], with a focus on inflammatory responses and cartilage breakdown. It is, however, important to realize that, e.g., Sox9 transcriptional activity is differentially regulated in healthy and OA chondrocytes [34], pointing to caution with generalized predictions. Regardless, the integration of the Keap1–Nrf2 system into multiple cellular signaling and metabolic pathways places Nrf2 activation as a critical regulatory node in pathophysiological conditions.

We then used Nrf2-KO mice to further verify our initial results in vivo and found that old Nrf2-KO animals had a decreased articular cartilage thickness in their knees. Moreover, immunohistochemical staining revealed less than half as many Sox9-positive chondrocytes in articular cartilage in knees from Nrf2-KO mice, compared with their WT littermates (*p* < 0.05) (Figure 5).

Importantly, Nrf2-KO mice revealed a mild osteoarthritic cartilage degeneration at an older age, while in young, mature animals, the articular cartilage of the knee still looked normal (Figure 6). Potentially progressively disturbed tissue homeostasis in aging mice with a chronic Nrf2 malfunction might explain our findings, as Sox9 still plays an essential role in the physiological control of cartilaginous tissues in even adult mice [28]. Given the important role of Sox9 for articular cartilage homeostasis, it is intriguing that we found significantly fewer Sox9-positive chondrocytes already in young, mature adult Nrf2-KO mice, compared with age-matched WT controls. A decreased expression of, among others, Nrf2 is in line with our findings pointing toward accumulated stresses in this tissue over time [35].

Remarkably, at least six phenotypes of OA were recently proposed [36]; among them is an aging-driven phenotype, against which physiological levels of Nrf2 activity seem to be protective. Under degenerative conditions, such as OA, alterations occur in mitochondrial structure dynamics and genome stability, resulting in reduced mitochondrial respiration and excessive production of ROS, thus leading to oxidative damage [36,37].

Increased OARSI OA scores were observed in old Nrf2-KO mice despite their cartilage looking normal at a young, mature age. Compared with healthy chondrocytes, reduced Nrf2 protein levels in human osteoarthritic cartilage were reported [35] but contradicted by another study reporting an increase in Nrf2 protein levels in human osteoarthritic cartilage, compared with non-osteoarthritic tissue [38]. Interestingly, the latter study reported significantly higher Nrf2 gene expression in macroscopically damaged osteoarthritic cartilage than in undamaged cartilage from the same OA patients [38]. In a mouse model of antibody-induced arthritis, we previously reported that Nrf2-KO mice had more severe cartilage injuries and more oxidative damage than WT mice [12]. Cai et al. also reported that Nrf2-KO mice revealed more severe cartilage damage, compared with WT mice, in two OA models—monosodium iodoacetate articular injection and destabilization of the medial meniscus [13]. We, therefore, postulate that Nrf2 may be essential to prevent age-dependent OA progression, potentially due to balancing antioxidant actions and maintaining cartilage homeostasis, especially in older adult life.

Notably, there is evidence of an age-associated imbalance between ROS production and antioxidant capacity in chondrocytes that seriously affects chondrocyte cell death or cartilage degradation [8]. In terms of longevity and the prevention of age-related diseases, how Nrf2 expression changes during the aging process is actually an important topic [39]. Age-related changes in Keap1–Nrf2 signaling have been reported in human skeletal muscle [40] and cardiac muscle or liver from aging rats [41,42]. OA is a classic age-related chronic degenerative disorder, and thus, it is likely that future pharmacological intervention with Nrf2 activators also holds tremendous potential for altering the course of OA. Moreover, Nrf2 has recently been reported to play a protective role in cartilage homeostasis during aging due to exposure to ROS. Importantly, overexpression of SOX9 was shown to alleviate the progression of human osteoarthritis in vitro and in vivo [43], which would be in line with our proposed working model. Upregulation of SOX9 further inhibits, among others, IL-1β-induced inflammatory response through Smad3.

OA is considered to be an “age-related low-grade inflammatory disease of the joints”, which causes the protective cartilage cushion between the bones to wear down, with an aberrant metabolism clearly contributing to it [36]. Notably, the metabolic demands of fully differentiated and quiescent chondrocytes are very different from chondrocytes in an inflammatory microenvironment.

TGF-β is crucially involved in the preservation of the chondrocyte phenotype under hypoxic conditions [44]. Further links between Nrf2 and the TGF-β superfamily signaling include the regulation of phosphorylation and thus stabilization of Sox9 protein in chondrocytes through p38 and Smad-dependent mechanisms [45]. To this end, the oxygenation level of the microenvironment may place the Keap1–Nrf2 system at the intersection between pathological and physiological processes in cartilage [46,47].

The pathogenesis and progression of OA further seem to be the result of the complex and dynamic interplay of mechanical, cellular, and systemic molecular factors [48]. Cellular homeostasis is, among others, maintained through heat shock protein (Hsp) 90, modulating the stability of their substrates, while activating the Keap1–Nrf2 pathway combats oxidative stress. As Hsp90 was linked to biomechanically induced OA in rats [49], general heat-shock-protein-mediated cell stresses may thus also contribute. It would, therefore, be interesting to study the intersection of both pathways in the future.

Within the different subtypes of OA, biomechanical alterations still dominate the risk for disease progression [36]. Biomechanical loading of articular cartilage is further directly linked to the interstitial osmotic pressure in the tissue [50]. The latter itself depends on proper proteoglycan production and ECM homeostasis, which, in turn, is largely controlled by Sox9. To this end, it is interesting that SOX9 was identified as a regulator of ADAMTSs-induced cartilage degeneration at the early stage of human OA [51]. The Keap1–Nrf2 pathway likely interacts with a plethora of physicochemical and biomechanical stimuli that control articular cartilage repair [52]. Interestingly, Sox9 is also a major driver behind the osmolarity-determined chondrogenic differentiation capacity of progenitor cells [53], and osmolarity improves chondrocyte ECM marker expression, but also specifically affects ADAMTS-4 and ADAMTS-5 [50]. One important function of Nrf2 could, therefore, be to maintain sufficiently high SOX9 expression in articular cartilage throughout aging, thereby mediating suppression of ADAMTSs to protect cartilage integrity and thus delay the onset of OA.

Notably, ROS-based regulation of Mitogen-activated protein kinase (MAPK) signaling cascades in human articular chondrocytes recently also pointed toward the complexity of Keap1–Nrf2 regulation [54]. Regulation of gene expression by histone acetyltransferases and histone deacetylases also drives the etiology of many age-related human diseases, including osteoarthritis [55,56], to which an imbalanced Nrf2 activity may contribute.

## 5. Conclusions

To the best of our knowledge, this is a first-time report indicating that the proximal upstream ARE site (ARE_2_) plays a crucial direct role in the Nrf2-mediated induction of the *SOX9* promoter in human chondrocytes. Importantly, we also provided evidence that a systemic Nrf2 deficiency results in Sox9 suppression in murine articular chondrocytes in vivo. At an older age, Nrf2-KO mice developed a mild osteoarthritic articular cartilage degeneration, which is in line with earlier reported proteoglycan loss upon postnatal Sox9 depletion [26]. Thus, we postulate that accumulative oxidative stress age-dependently contributes to cartilage degeneration in the knee and that Nrf2 helps protect its ECM integrity through maintaining Sox9-mediated postnatal tissue homeostasis. Future pharmacological Nrf2 stimulating therapies may hold the potential to alter the course of primary wear-and-tear OA to this end.

## Figures and Tables

**Table 1:** Comparison of OA score in young mature adult and old mice.

OASRI Grade	Histological Findings (Safranin-O Staining)	Young Mature Adult		Old	
		Nrf2-WT (*n* = 6)	Nrf2-KO (*n* = 5)	Nrf2-WT (*n* = 11)	Nrf2-KO (*n* = 8)
		Male 2 Female 4	Female 5	Male 5 Female 6	Male 4 Female 4
0	Normal cartilage	6	5	2	−
0.5	Loss of staining without structural changes	−	−	8	2
1	Small fibrillations without cartilage loss	−	−	1	3
2	Vertical clefts and/or erosion down to the layer just below the surface and some loss of surface lamina	−	−	−	3
3	Extension of vertical tears and/or erosions to the calcified cartilage (<25%)	−	−	−	−
4	Extension of vertical tears and/or erosions to the calcified cartilage (25−50%)	−	−	−	−
5	Extension of vertical tears/erosions to the calcified cartilage (50−75%)	−	−	−	−
6	Extension of vertical tears/erosions to the calcified cartilage (>75%)	−	−	−	−

OARSI, the Osteoarthritis Research Society International.

## Data Availability

Data are contained within the article.
